# Closing Editorial: Toward Safe, Equitable, and Intelligent Healthcare

**DOI:** 10.3390/healthcare14172703

**Published:** 2026-08-24

**Authors:** Di Tang, Thomas Yuen Tung Lam

**Affiliations:** 1 The Nethersole School of Nursing, Faculty of Medicine, The Chinese University of Hong Kong, Hong Kong SAR, China; dixontang@cuhk.edu.hk; 2 Stanley Ho Big Data Decision Analytics Research Centre, The Chinese University of Hong Kong, Hong Kong SAR, China

## 1. Introduction

In recent years, the rapid evolution of artificial intelligence (AI) technologies has increasingly transformed the landscape of global healthcare delivery [1,2]. Today, in 2026, AI is no longer just a trending topic and is now starting to be integrated into real-world clinical workflows [3,4]. This transition highlights the great potential of AI in enhancing diagnostic precision and optimizing medical resource allocation [5,6,7], while simultaneously exposing persistent challenges in ethics, regulatory frameworks, and health equity [8,9,10]. This Special Issue of Healthcare brings together a diverse collection of 17 research and review articles, offering a comprehensive exploration of AI’s multidimensional role in transforming healthcare across different populations, clinical scenarios, and geographical regions.

The 17 contributions (Contributions 1–17) included in this Special Issue collectively address the critical dimensions of medical AI implementation: from the clinical application of Large Language Models (LLMs) and the accuracy of predictive analytics, to clinician acceptance, legal and ethical considerations, and global health equity. Understanding these diverse applications is essential, as AI is rapidly evolving from a standalone supplementary tool into an indispensable, core component of sustainable healthcare delivery systems. Table 1 lists all the contributions in the Special Issue.

## 2. The Rise of LLMs in Medical Applications

Understanding the capabilities of LLMs in complex medical decision-making forms the foundation of current clinical AI integration. Siebielec et al. (Contribution 1) assessed the performance of ChatGPT-3.5 in the Polish Medical Final Examination, revealing an average accuracy of approximately 60%, which remained lower than that of human examinees. This highlights that while LLMs possess foundational medical knowledge, their outputs must be cross-verified. Furthermore, AI’s application in specialized fields has also been explored. Kamal (Contribution 2) analyzed the accuracy of AI chatbots in pediatric orthopedics, while another study (Contribution 3) demonstrated ChatGPT-4.0’s high concordance (76.4%) in multidisciplinary tumor boards (MDTs). These patient- and clinician-centric analyses emphasize that while LLMs provide efficient information retrieval, they cannot replace clinical intuition. A “human-in-the-loop” approach remains essential [1,11]. Beyond clinical accuracy, the “warmth” of LLMs in patient communication is equally important. A study on narrative-based prompting (Contribution 4) showed that LLMs (like GPT-o3-mini) can be guided to significantly improve empathy, personalization, and clarity in patient-facing communication, offering a potential strategy to mitigate “algorithm aversion” [12].

## 3. Predictive Analytics and Precision Medicine

Multiple contributions demonstrate the remarkable efficacy of AI predictive models in specific disease management. An innovative cardiovascular disease prediction model, HeartEnsembleNet (Contribution 5), utilized a hybrid ensemble learning approach to significantly improve screening accuracy (92.95%). Similarly, generative AI and deep learning have shown immense value in early warning and management of chronic complications, such as diabetic foot ulcers (Contribution 6), and in enhancing diagnostic precision in dental imaging (Contribution 7). Broadening the scope, a bibliometric analysis (Contribution 8) highlighted the exponential growth of machine learning in primary health care for early diagnosis. Furthermore, a systematic review of reinforcement learning (Contribution 9) showcased its potential in creating dynamic treatment regimes for precision medicine. Together, these studies prove that AI-driven predictive analytics effectively facilitate early screening and treatment, aligning perfectly with the global shift towards preventive healthcare [1,13,14].

## 4. Ethical, Legal, and Regulatory Frameworks

As the clinical application of AI deepens, the lag in regulatory and legal frameworks becomes increasingly apparent [15,16]. A comprehensive review of AI-based Software as a Medical Device (AI-SaMD) (Contribution 10) pointed out that traditional static approval pathways struggle to accommodate continuously learning algorithms, calling for dynamic regulatory mechanisms. Meanwhile, a mixed-methods study in Saudi Arabia (Contribution 11) investigated clinicians’ legal concerns, revealing that unclear liability (i.e., “who is responsible if AI makes a mistake”) and data privacy are major barriers to adoption. To address these data privacy and security challenges, federated learning emerges as a crucial technical solution. Another review article (Contribution 12) highlighted how federated learning enables collaborative machine learning across institutions while preserving patient privacy and meeting stringent regulatory standards.

## 5. Global Disparities and Adoption Barriers

A critical theme emerging from this Special Issue is health equity and the “digital divide”. A registry-based analysis (Contribution 13) revealed a severe global imbalance in AI clinical trials, with extreme geographic concentration undermining global health equity and algorithm generalizability. In terms of adoption barriers, a qualitative study based on Normalization Process Theory (Contribution 14) explored the use of AI agents among older patients with chronic obstructive pulmonary disease (COPD), identifying trust deficiency, technical anxiety, and blurred human–machine boundaries as significant hurdles. These barriers are not limited to patients. Healthcare providers also face challenges. An observational study (Contribution 15) found that while physical therapists are eager to adopt AI, a lack of formal training and organizational preparedness hinders implementation. Similarly, a review of AI in telepsychiatry (Contribution 16) underscored the disconnect between rapid technological evolution and the institutional maturity required for clinical integration. Finally, a topic modeling study of Korean medical AI research (Contribution 17) perfectly summarized this transition: the field is rapidly shifting from algorithm-centric studies to a more interpretable, integrated, and human-centered paradigm.

## 6. Critical Research Gaps and Future Priorities

Although this Special Issue has advanced our understanding of AI implementation in healthcare, several research gaps in the field remain to be filled. A primary challenge is the current severe lack of prospective, real-world studies [17]. Most existing AI research still relies on retrospective datasets, creating a need for more long-term, prospective randomized controlled trials (RCTs) to accurately evaluate the impact of AI interventions on long-term clinical endpoints [17,18]. Furthermore, the “black-box” nature of AI models remains a substantial barrier to building clinical trust, highlighting the pressing need for explainable AI (XAI) [19]. Future research must focus on developing transparent models capable of clearly articulating their reasoning processes to clinicians [20,21]. Meanwhile, comprehensive health economic evaluations are also necessary. To provide solid data support for policymaking and clinical adoption, standardized evaluation frameworks must be established to comprehensively measure the overall societal costs, physicians’ time saved, and long-term cost-effectiveness following the integration of AI systems [22,23,24].

## 7. Implications for Policy and Practice

The findings presented in this Special Issue offer practical guidance for policymakers and healthcare administrators. First, regulatory bodies must accelerate the development of globally harmonized standards for the approval and post-market surveillance of AI-SaMD [15,25]. Second, medical education must be reformed to integrate “AI literacy” and foundational data science into core curricula, preparing future professionals for hybrid medical practice [26,27]. Finally, health equity must be positioned at the core of AI research and development, with mandatory requirements for validating AI models across diverse population datasets [8,28,29].

## 8. Conclusions

At the current stage, medical AI has transitioned from proving “what AI can do” to addressing “how to use AI safely and equitably in clinical settings”. By examining the application of AI across diverse clinical scenarios, populations, and regulatory landscapes, this Special Issue provides valuable evidence for this transition. A hybrid care model, one that leverages the computational advantages of AI while preserving the empathy and comprehensive judgment of human clinicians, represents a viable pathway for future healthcare delivery. The research gaps and challenges identified in this Special Issue present opportunities for the scientific community to further advance healthcare digitalization. Ultimately, the goal is to provide safer, more efficient, and equitable medical care for all patients in an increasingly intelligent healthcare environment.

## Figures and Tables

**Table 1:** List of all the contributions in the Special Issue.

Number	Articles
Contribution 1	Siebielec, J.; Ordak, M.; Oskroba, A.; Dworakowska, A.; Bujalska-Zadrozny, M. Assessment Study of ChatGPT-3.5’s Performance on the Final Polish Medical Examination: Accuracy in Answering 980 Questions. Healthcare 2024, 12, 1637.
Contribution 2	Kamal, A.H. AI Chatbots in Pediatric Orthopedics: How Accurate Are Their Answers to Parents’ Questions on Bowlegs and Knock Knees? Healthcare 2025, 13, 1271.
Contribution 3	Dogan, I.; Bartin, M.K.; Sonmez, E.; Seyran, E.; Bozkurt, H.A.; Yuksek, M.; Serbes, E.D.; Zalova, G.; Celik, S. Chat GPT Performance in Multi-Disciplinary Boards—Should AI Be a Member of Cancer Boards? Healthcare 2025, 13, 2254.
Contribution 4	Wang, F.; Wang, N.; Xu, W.; Zhang, P. Unlikely Storyteller: Leveraging Narrative-Based Communication in LLM-Generated Medical Advice. Healthcare 2026, 14, 1015.
Contribution 5	Zaidi, S.A.J.; Ghafoor, A.; Kim, J.; Abbas, Z.; Lee, S.W. HeartEnsembleNet: An Innovative Hybrid Ensemble Learning Approach for Cardiovascular Risk Prediction. Healthcare 2025, 13, 507.
Contribution 6	Alkhalefah, S.; AlTuraiki, I.; Altwaijry, N. Advancing Diabetic Foot Ulcer Care: AI and Generative AI Approaches for Classification, Prediction, Segmentation, and Detection. Healthcare 2025, 13, 648.
Contribution 7	Khattak, O.; Hashem, A.S.; Alqarni, M.S.; Almufarrij, R.A.S.; Siddiqui, A.Y.; Anis, R.; Ahmad, S.; Fareed, M.A.; Alothmani, O.S.; Alkhershawy, L.H.S.; et al. Deep Learning Applications in Dental Image-Based Diagnostics: A Systematic Review. Healthcare 2025, 13, 1466.
Contribution 8	Završnik, J.; Kokol, P.; Žlahtič, B.; Blažun Vošner, H. Machine Learning in Primary Health Care: The Research Landscape. Healthcare 2025, 13, 1629.
Contribution 9	Frommeyer, T.C.; Gilbert, M.M.; Fursmidt, R.M.; Park, Y.; Khouzam, J.P.; Brittain, G.V.; Frommeyer, D.P.; Bett, E.S.; Bihl, T.J. Reinforcement Learning and Its Clinical Applications Within Healthcare: A Systematic Review of Precision Medicine and Dynamic Treatment Regimes. Healthcare 2025, 13, 1752.
Contribution 10	Ebad, S.A.; Alhashmi, A.; Amara, M.; Miled, A.B.; Saqib, M. Artificial Intelligence-Based Software as a Medical Device (AI-SaMD): A Systematic Review. Healthcare 2025, 13, 817.
Contribution 11	Alanazi, A. Assessing Clinicians’ Legal Concerns and the Need for a Regulatory Framework for AI in Healthcare: A Mixed-Methods Study. Healthcare 2025, 13, 1487.
Contribution 12	Abbas, S.R.; Abbas, Z.; Zahir, A.; Lee, S.W. Federated Learning in Smart Healthcare: A Comprehensive Review on Privacy, Security, and Predictive Analytics with IoT Integration. Healthcare 2024, 12, 2587.
Contribution 13	Kwon, C.-Y. Beyond the Growth: A Registry-Based Analysis of Global Imbalances in Artificial Intelligence Clinical Trials. Healthcare 2025, 13, 2018.
Contribution 14	Cui, S.; Wang, S.; Deng, J.; Jia, R.; Jiang, Y. Facilitators and Barriers of Using an Artificial Intelligence Agent in Chronic Disease Management: A Normalization Process Theory-Guided Qualitative Study of Older Patients with COPD. Healthcare 2026, 14, 268.
Contribution 15	Aldhahi, M.I.; Alorainy, A.I.; Abuzaid, M.M.; Gareeballah, A.; Alsubaie, N.F.; Alshamary, A.S.; Hamd, Z.Y. Adoption of Artificial Intelligence in Rehabilitation: Perceptions, Knowledge, and Challenges Among Healthcare Providers. Healthcare 2025, 13, 350.
Contribution 16	Bobkov, A.; Cheng, F.; Xu, J.; Bobkova, T.; Deng, F.; He, J.; Jiang, X.; Khuzin, D.; Kang, Z. Telepsychiatry and Artificial Intelligence: A Structured Review of Emerging Approaches to Accessible Psychiatric Care. Healthcare 2025, 13, 1348.
Contribution 17	Yun, H.; Lee, Y. Mapping the Landscape of Medical AI Research in Korea Using Topic Modeling. Healthcare 2026, 14, 549.
